# Multi-endpoint assessment of tunnel wash water and tyre-particle leachate in zebrafish larvae

**DOI:** 10.1016/j.toxrep.2025.102096

**Published:** 2025-07-23

**Authors:** Shubham Varshney, Chinmayi Ramaghatta, Prabhugouda Siriyappagouder, Andy M. Booth, Lisbet Sørensen, Pål A. Olsvik

**Affiliations:** a Faculty of Biosciences and Aquaculture, Nord University, Bodø, Norway; b SINTEF Ocean, Trondheim, Norway

**Keywords:** Tyre-wear pollution, Tunnel wash runoff, Tyre leachate

## Abstract

Washing of road tunnels is essential for removing accumulated pollutants such as tyre wear particles, brake dust, exhaust residues, and road debris to ensure visibility and safe driving. Tunnel washing generates large volumes of contaminated runoff known as untreated tunnel wash runoff (UTWR). Some countries filter UTWR through a sedimentation process before release to reduce contamination, generating what is known as treated tunnel wash runoff (TWR). This study investigates the potential environmental impact of diluted UTWR (25 %) and TWR (50 %) by evaluating their toxicity in fish and comparing the effect to tyre-particle leachate (TPL, 2 g/L). UTWR was collected during tunnel cleaning, and TWR was collected after 14 days of filtration through sand sediments, from the Bodø tunnel in Norway. Zebrafish larvae, used as a fish model, exposed to contaminated runoff exhibited increased mortality, impaired growth, developmental anomalies, altered swimming behaviour, and changes in gene expression. Both UTWR and TWR exposure induced significant toxicity in zebrafish larvae, though the toxicity caused by TWR was notably lower than that of UTWR. This study shows that current filtration methods of tunnel wash water reduce the levels of most pollutants, however, more research is needed on how tunnel wash-water runoff affect aquatic ecosystems.

## Highlights

• High levels of heavy metals, PAHs and 6PPDq found in untreated tunnel wash runoff.
• Tunnel wash runoff and tyre-particle leachate increased oxygen consumption.
• All chemical mixtures reduced larval swimming.
• Untreated tunnel wash runoff has higher toxicity compared to treated tunnel runoff.

## Graphical Abstract

**Figure:**
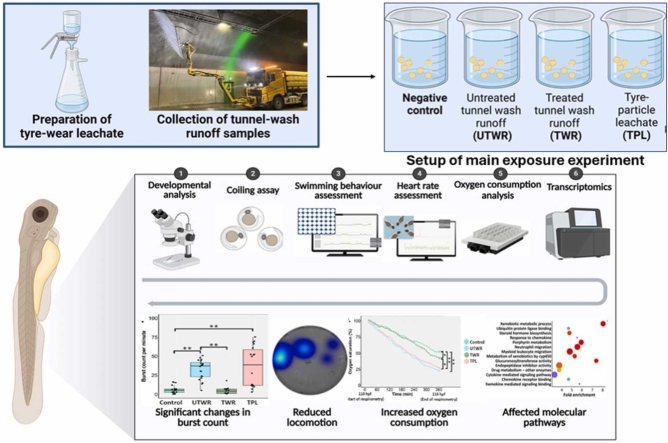

## 1 Introduction

The rapid expansion of urban infrastructure has resulted in a global road network of over 21 million km, with projections indicating an additional 3–4.7 million km by 2050 [21], [32]. This extensive increase has significantly contributed to increased environmental contaminants like road and tyre-wear pollutants (particles and chemicals), brake dust, vehicle exhaust, and oil spills [11], [16]. Deposition of tyre-wear pollutants, vehicular exhaust, sand and dust in tunnels create chemical and particulate pollution that poses significant safety risks, reduces lighting quality, and impairs visibility [30], [10]. Hence, tunnels are regularly washed with water and soap, depending on vehicular traffic volume and geographical location [12], [37]. The runoff generated, known as tunnel wash runoff, is typically rich in pollutants such as zinc (Zn), lead (Pb), copper (Cu), polycyclic aromatic hydrocarbons (PAHs), and abrasion particles from brakes, tyres, and the road surface [51]. Abrasion particles are macro- and microscopic particles generated through mechanical wear of tyres, brakes, and road surfaces during vehicle movement [42]. Most countries either do not treat this tunnel runoff at all (untreated tunnel wash runoff; UTWR) or, at most, subject it to basic sedimentation processes (treated tunnel wash runoff; TWR) before discharging it into the environment [34]. For this reason, tunnels are often considered hotspots for pollutant accumulation [1]. Additionally, the toxicity of UTWR and TWR is influenced strongly by the chemical composition of the runoff, which further depends on contaminant load, vehicular traffic, temperature, tyre composition, driving pattern and environmental conditions [19], [10].

Tyre particle leachate (TPL) is a complex mixture of organic and inorganic chemical pollutants generated through chemical desorption from vehicle tyres into the surrounding water. These chemicals include heavy metals, such as Zn, and PAHs, which are recognized for their carcinogenic potential [13]. Additionally, the leachate contains vulcanizing agents and antioxidants, including N-(1,3-Dimethylbutyl)-N′-phenyl-p-phenylenediamine (6PPD). The addition of 6PPD (0.4–2 %) extends the lifespan of tyres as it prevents the styrene butadiene rubber from breakdown caused by ozone, heat, and other environmental stressors [15], [17]. When tyres wear down, 6PPD is released into the environment, where it reacts with ozone and forms a highly toxic compound known as 6PPD-quinone (6PPDq) [47]. 6PPDq has been linked to mass mortality events in coho salmon (*Oncorhynchus kisutch*) in the United States [47], and recent studies have demonstrated that this compound is neurotoxic, impairing neuronal function and altering swimming behaviour in multiple fish species [25], [49], [50].

Norway has an extensive network of tunnels with an approximated total length of 800 km [37]. These tunnels are mechanically washed with high-pressure water and 0.5–1 % soap or detergents [34]. Tunnel washing can be categorized into two main types based on the extent of cleaning: half wash and full wash [38]. A half-wash is usually conducted monthly and involves cleaning the road surface and the tunnel walls up to a height of three meter [51]. In contrast, a full wash is performed every three to six months and encompasses thorough cleaning of the road surface, the entire height of the walls, and the tunnel roof [51]. Routine tunnel cleaning generates vast amounts of wash runoff discharged to surface water and groundwater. It is estimated that both full washes and half washes produce approximately 100 L of runoff per meter in a tunnel [51]. This can be illustrated with for example the Bodø Tunnel, a 2.8-km long, 4-lane tunnel that undergoes monthly washing, resulting in an annual runoff of approximately 1200 m³ (1.2 million litres). In Norway, this runoff is usually filtered through a sedimentation process before being released into the environment [33], [35], [43]. This process reduces the levels of contaminants but does not eliminate them entirely, leaving some pollutants that could still impact the environment.

There is a substantial scientific gap on knowledge about the toxicity of tunnel-wash water and tyre-derived chemical contaminants. Further research is needed on whether current filtration methods, primarily sedimentation, are efficient and reduce the levels of pollutants and their associated toxicity adequately before the wash-water is released into the environment. Hence, the aim of this study was to compare the toxicity of treated versus untreated tunnel wash-water runoff. TPL was included in the assessment to represent the chemical mixture of vehicle tyres. Assessing the toxicity of TPL alongside UTWR and TWR in a single study enables a comparative assessment of tyre-derived pollutants and the effectiveness of the tunnel wash-water treatment process.

## 2 Materials and methods

### 2.1 Collection of treated and untreated tunnel runoff samples

**Fig. 1:**
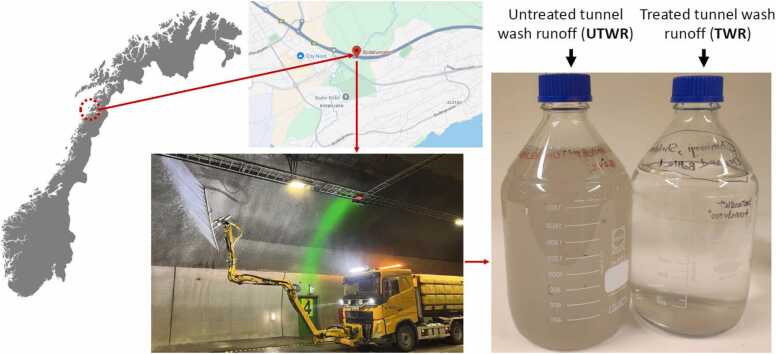
Geographic location of the 2.8 km long Bodø Tunnel in Nordland, Norway (top left). The bottom center image shows the tunnel cleaning process using a high-pressure water jet system. Samples of tunnel wash water were collected before and after treatment, shown in the laboratory bottles (right most). Photo Grete Livik, Statens Vegvesen.

The Bodø Tunnel, situated in Bodø city in Northern Norway, is a 4-lane, 2.8-km long tunnel with 15 600 vehicles passing each day in 2023. Tunnel wash water samples were collected at the Bodø Tunnel in April 2023 after a half-wash cleaning (Fig. 1). Untreated wash runoff (UTWR) was collected in the tunnel during cleaning directly in the drainage system. To prevent environmental pollution, the Norwegian Public Roads Administration (Statens Vegvesen) filter the runoff through sedimentation ponds before it is released through the municipality pipes and into nature. TWR was collected after filtration through sediments for 14 days. We collected UTWR and TWR samples in three replicates for this study after one tunnel half-wash procedure (Fig. 1).

### 2.2 Preparation of tyre-particle leachate (TPL)

Crumb rubber produced from end-of-life vehicle tyres was obtained from a recycling facility and used to prepare the TPL according to previously reported methods [14], [3]. Crumb rubber samples were added to E3 media in glass bottles wrapped in aluminium foil to prevent photoreaction. The bottles were shaken in the dark at 28°C on a horizontal stirrer (150 rpm) for seven days. Afterward, the solution was filtered using a sterile 0.2 μm VWR vacuum filtration system (Cat. No. 87006–066, VWR International) to separate the leachate from the tyre particles. A stock solution of 10 g/L TPL was prepared through this protocol and stored in the dark at 5°C until further use.

### 2.3 Chemical analysis of UTWR, TWR and TPL samples

The E3 media (negative control), UTWR, TWR and TPL samples were analysed the for presence of heavy metals, polychlorinated biphenyls (PCBs), polycyclic aromatic hydrocarbons (PAHs), volatile organic compounds (VOCs), total petroleum hydrocarbons (TPHs) and aliphatic compounds by an accredited laboratory (ALS, Oslo, Norway). For the analysis of 6PPDq, analysis was performed by the Norwegian Veterinary Institute (Ås, Norway).

### 2.4 Zebrafish husbandry, breeding and embryo collection

Adult zebrafish of the AB strain were bred and maintained in a recirculatory system (Aquatic Habitats Z-Hab System, MBK Installations Ltd., United Kingdom) at the Mørkvedbukta Research Station, Nord University (Bodø campus). They were kept in optimal conditions, with a water temperature of 28 ± 2°C, pH between 7 and 8, conductivity at 200–300 μS/cm, hardness at 100–200 ppm and a photoperiod of 14 L:10D. The adult zebrafish were fed with Zebrafeed® micro diet (Sparos Lda, Olhão, Portugal) twice a day along with a live feed of rotifers a day. The adult zebrafish (1: 1; male: female) were bred in the breeding tanks to obtain eggs. Fertilized eggs were separated from the unfertilized eggs using a binocular microscope (Leica Z00M 2000, Leica, Buffalo, NY, USA). Viable eggs were kept in the autoclaved E3 media and reared in a climate chamber (Sanyo MIR-154, Sanyo Scientific, Bensenville, Illinois, USA) at 28 ± 1℃. According to European legislation [8], zebrafish embryos and larvae under 120 h old are not classified as protected animals, so no special authorization is needed for experiments involving them (EC, 2010). The exposure experiments were terminated at 118 hpf (hours post fertilisation), and larvae were euthanised by immersing in water containing 200 mg/L of MS222 along with 200 mg/L of sodium bicarbonate [31].

### 2.5 Preliminary mortality assessment of zebrafish eggs in response to TPL and UTWR samples

A preliminary mortality assessment of zebrafish eggs was conducted in UTWR and TPL samples to decide on exposure concentrations. The rationale was to evaluate the initial toxic effects and establish sublethal concentration ranges of UTWR and TPL before the main exposure experiment. This step was essential to avoid selecting concentrations that might cause excessive mortality, ensuring more consistent and interpretable results in the primary experiment. We exposed 2 hpf zebrafish eggs (n = 20) to UTWR (25, 50 and 75 % concentrations) and TPL (1, 2 and 5 g/L). The 96 h exposures were performed in 250 mL glass beakers, and three replicates. Partial semi-static exposure regime was followed, with ∼90 % of the exposure media replaced every 24 h. The rationale behind partial medium renewal was to prevent air exposure that could induce desiccation stress and handling-related injuries or mortalities. The exposure beakers with eggs were kept in a climate chamber (Sanyo MIR-154, Sanyo Scientific, Bensenville, Illinois, United States of America) at 28 ± 1°C with a 14 L:10D photoperiod cycle. During the whole exposure period, the dead eggs/embryos were carefully discarded without disturbing the remaining animals, and mortalities in every beaker were assessed every 24 h.

### 2.6 Experimental design for exposure experiments

Four exposure groups were used for the main exposure experiments: (i) negative control (1X E3 media), (ii) UTWR (25 % concentrate), (iii) TWR (50 % concentrate), and (iv) TPL (2 g/L). For UTWR, 25 % (2 g/L) was selected after pre-testing which showed that exposure to higher concentration of UTWR (50 and 75 %) caused significant mortality. For TWR, a 50 % dilution was selected based on consideration that TWR had already undergone sedimentation-based treatment, it was anticipated to contain significantly lower levels of contaminants, as confirmed by our chemical analyses. After further pre-testing, a TPL exposure concentration of 5 g/L was selected. Overall, in all exposure groups lethal doses were avoided as it could compromise the ability to assess sublethal endpoints such as behaviour, development and gene expression. Also, we followed the OECD guidelines (Test No. 236: Fish Embryo Acute Toxicity (FET) Test) with slight modification [36]. The pH of all exposure solutions was maintained between 7.2 and 7.4. All exposure experiments were conducted in triplicates. Glass beakers were pre-treated with their respective test solutions for 24 h before the experiment. Zebrafish embryos (n = 40) at 2 hpf were exposed to a 40 mL test solution (1 mL test solution per embryo) in a 250 mL glass beaker for 96 hrs. The beakers were covered with parafilm (with several holes for the oxygen flow) to minimize the evaporation of the test medium. The glass beakers were kept in a climate chamber at 27±1 °C (Sanyo MIR-154, Sanyo Scientific, Bensenville, Illinois, United States of America) with 14 L:10D photo-period cycles. To replenish the concentration of contaminants in the test solution, approximately 80–90 % of the solution was replaced every 24 h. Complete or 100 % renewal of test solutions were avoided to minimize embryo stress and prevent potential desiccation during the transfer. At the end of 96 h of exposure, test solutions were replaced with E3 medium (up to 116 hpf). The larvae were then assessed for various endpoints: development, early embryonic movement, swimming behaviour, heart rate, oxygen saturation and transcriptomic responses.

### 2.7 Developmental analysis

Zebrafish eggs/larvae (n = 10 per treatment) at different developmental stages (24, 48, 72, 96 and 116 hpf) were taken to study the morphological characters using an inverted stereomicroscope Olympus SZX12 (Melville, USA) equipped with an Olympus SC50 (Olympus soft imaging solutions, Münster, Germany) video camera. Individual eggs or larvae were placed on a cavity glass slide (Cat. No. MARI1216520, VWR), and 3.5 % methylcellulose was applied to immobilize the larvae. The raw images of eggs/larvae were analysed for body length, eye size, swim bladder size and yolk size using the DanioScope™ software v1.1 (Noldus Information Technology, Netherlands). Head-to-trunk angles of the 96 and 116 hpf larvae were analysed using ImageJ software (http://imagej.net).

### 2.8 Early embryonic movement assessment/ Coiling assay

The coiling assay or spontaneous tail movement assay indicates how embryonic random movements develop into intricate behavioural patterns in larval or adult stages [52]. The assay was performed following the methodology of Zindler et al. [58]. At 24 hpf, zebrafish embryos (n = 20 per treatment) were placed in glass petri dishes for recording under an Olympus SZX12 inverted stereomicroscope (Melville, USA) equipped with an Olympus SC50 video camera (Olympus Soft Imaging Solutions, Münster, Germany). Tail coiling activity was recorded for 3 min, and the raw videos were evaluated for burst count, inactivity and burst duration using the DanioScope™ *v*1.1 software (Noldus, Wageningen, Netherlands). Burst count per min means the number of larval contractions observed in an embryo at a particular developmental stage and is an indicator of the neuromuscular development of the embryo [56]. Inactivity duration is when an embryo shows no spontaneous movement [56]. Assessment of this inactivity duration is crucial as it indicates the balance between the active and resting states of the developing embryo. More extended periods of inactivity may indicate developmental delay or neuromuscular coordination problems, while shorter periods may indicate overactivity or abnormal neurodevelopment [45].

### 2.9 Swimming behaviour assessment

Zebrafish larvae (n = 20 per treatment) at 116 hpf were observed for their swimming patterns using the DanioVision system (Noldus Information Technology, Netherlands) in a 24-well glass plate. The assessment of swimming behaviour was conducted based on the methodology described in our previously published study [48]. In brief, 20-minute recordings (two alternate cycles, each of five minutes light and dark) were performed at 28 ± 1 °C in the DanioVision observation chamber (Noldus Information Technology, Netherlands). The behavioural video recordings were analysed using EthoVision XT *v*16.0 software (Noldus Information Technology, Netherlands). Subsequently, locomotory heatmaps and trajectory maps were generated using EthoVision® XT *v*16.0 software.

### 2.10 Heart rate assessment

Zebrafish larvae (n = 10 per treatment) at 116 hpf were observed for heart rate assessment, following the protocol outlined in our previously published study [48]. Before video recording the heart rate the larvae were acclimatised to recording conditions by keeping them in dark for 20 min at 28 ± 1°C. Following this, larvae were immobilized in 3.5 % methylcellulose, and 30-second video recordings were performed at 45x magnification using an Olympus SZX12 stereomicroscope (Melville, USA) equipped with an Olympus SC50 video camera (Olympus Soft Imaging Solutions, Münster, Germany). The DanioScope™ software v1.1 (Noldus Information Technology, Netherlands) extracted the heart rate from raw videos.

### 2.11 Oxygen consumption assessment

The oxygen consumption rate of 110 hpf zebrafish larvae (n = 12 per treatment) was measured following the protocol outlined in our previously published study [48]. Briefly, larvae were kept in an air-tight 24-well plate sensor dish for oxygen consumption assessment in a microplate-based respirometry system (Loligo® Systems, Viborg, Denmark) maintained at 28 ± 1°C. The oxygen consumption rate was recorded for six hours using the software program MicroResp® version 1.0.4 (Loligo Systems, Viborg, Denmark).

### 2.12 RNA sequencing methodology

The whole larval transcriptome analysis of 116 hpf zebrafish larvae (n = 5 per treatment) was conducted following the protocol detailed in our previously published study [48]. In short, the total RNA was isolated using the Direct-zol RNA MiniPrep (Cat. No. R2052, Zymo Research, CA, USA) kit. The quality assessment and quantification of isolated RNA was performed using the TapeStation 4150 (Cat. No. G2964AA, Agilent Technologies, Santa Clara, CA, USA) and Qubit™ 4 Fluorometer (Cat. No. Q33238, Thermo Fisher Scientific, Waltham, MA, USA) respectively. Only high-quality RNA (RIN value >8.5) was further taken for the library preparation. RNA-seq libraries were prepared using the NEBNext Ultra™ RNA Library Prep Kit (Cat. No. E7760S, NE Biolabs, Ipswich, MA, USA) and the Poly(A) mRNA Magnetic Isolation Module (Cat. No. E74490S, NE Biolabs), following the manufacturer’s protocol. Sequencing (75 bp, single end) was conducted using the Illumina NextSeq 500 sequencer (Cat. No. FC-404–2005, Illumina, San Diego, CA, USA) with the NextSeq 500/550 High Output *v*2 kit at the high throughput sequencing facility at Nord University (Bodø, Norway).

### 2.13 Bioinformatic analysis of RNA-seq data

The raw sequencing reads were filtered using the fastp software in a Linux environment to remove low-quality sequences (Phred score < 30), ensuring that only high-quality reads (Phred score > 30) were retained for downstream processing [5]. Further bioinformatics analysis, such as mapping, normalization of reads and differential gene expression through DESeq2 software to obtain differentially expressed genes (DEGs), as detailed in previous publications [20], [23], [27]. From here on, "DEGs" refers to transcripts with a Log2 fold change greater than + 1 or less than −1 and a p-adjusted value below 0.05, determined using the Benjamini-Hochberg method for multiple test correction. Gene Ontology (GO) and Kyoto Encyclopedia of Genes and Genomes (KEGG) pathway analysis were performed using DAVID *v*2023q4. GO terms or KEGG pathways with a false discovery rate (FDR) below 0.05 were deemed statistically enriched.

### 2.14 Statistical analysis

All data, excluding the RNA-seq data, were first tested for normality using the Kolmogorov-Smirnov and Shapiro-Wilk tests. For data that followed a normal distribution (development, heart rate, non-angular behaviour), one-way ANOVA was conducted, followed by Dunnett's multiple comparison tests to assess the effects of the treatment. Furthermore, circular data (angular behavioural) was analysed using the Rayleigh test (test of uniformity) followed by Stephens Modified Watson's test. Respiration data was analysed using two-way ANOVA (factor 1: treatment, factor 2: time), followed by Tukey's HSD. All statistical analyses and data visualisations were performed using R studio *v*2023.12.1. A p-value of less than 0.05 was considered statistically significant (*), while a p-value of less than 0.005 was regarded as highly significant (**).

## 3 Results

### 3.1 Chemical characterization of tyre-wear pollutants

Elevated levels of heavy metals such as Fe (865 µg/L), Zn (35 µg/L), Cu (10.3 µg/L) and Ni (3.57 µg/L) were found in UTWR water (Supplementary Table 1). The concentrations of most of them were reduced in TWR water (Fe: 21.8 µg/L, Zn: 17.7 µg/L and Cu: 8.88 µg/L) except for Ni, whose concentration was considerably higher in TWR water (10.6 µg/L) than in UTWR water (3.57 µg/L). In the TPL sample, considerable amounts of heavy metals (Cd: 0.08 µg/L, Cu: 1.87 µg/L and Ni: 1.85 µg/L) were found, but concentrations of most of them were lower when compared to both UTWR and TWR samples. The exception was for Zn, where the highest level was quantified in the TPL sample (6270 µg/L). A broad range of aromatic and aliphatic hydrocarbons were quantified in both UTWR and TPL leachates, but with different distributions in each sample type. In TPL, lower molecular mass aromatic compounds like benzene (0.64 µg/L), toluene (19.7 µg/L) naphthalene (0.093 µg/L), acenaphthene (0.076 µg/L) and fluorene (0.02 µg/L) were detected, while higher molecular mass compounds like phenanthrene, fluoranthene, pyrene and benzo[*ghi*]perylene were quantified in UTWR (Supplementary Table 1). 6PPDq was found at a concentration of 27.6 µg/L in UTWR water but was 1800-fold lower in TWR water (0.05 µg/L) (Supplementary Table 1). The concentration of 6PPDq in TPL was similar to that of TWR water (Supplementary Table 1).

### 3.2 UTWR and TPL exposure caused mortality

**Fig. 2:**
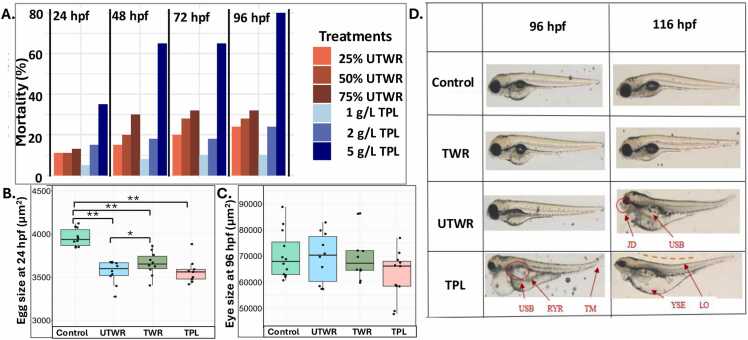
Effect of tyre-wear pollutants on the developmental indices of zebrafish larvae. **A)** Mortality of zebrafish eggs and larvae exposed to UTWR (25, 50 and 75 %) and TWL (1, 2 and 5 g/L) at 24, 48, 72 and 96 hpf, **B)** egg size at 24 hpf, **C)** eye size at 96 hpf and **D)** developmental abnormalities at 96 hpf and 116 hpf, respectively. PE = pericardial edema, JD = jaw deformity, USB = uninflated swim bladder, LO = lordosis, RE = reduced eye, YSE = yolk sac edema and RYR = reduced yolk resorption. Statistical significance is indicated by * (p < 0.05) and ** (p < 0.005).

The mortality of zebrafish embryos and larvae increased with higher concentrations of UTWR and TPL across all time points. At 24 hpf, the mortality of zebrafish embryos ranged from 11–13 % for UTWR and 5–35 % for TPL (Fig. 2**A;** Supplementary Table 2). Similarly, at 96 hpf, the mortality of zebrafish larvae reached 24–32 % for UTWR and 10–80 % for TPL (Fig. 2**A;** Supplementary Table 2). Higher concentrations, particularly in TPL treatments, resulted in significantly greater mortality, highlighting the toxic effects of the soluble wash water components. (Fig. 2**A;** Supplementary Table 2).

### 3.3 Growth and development at different developmental stages

**Fig. 3:**
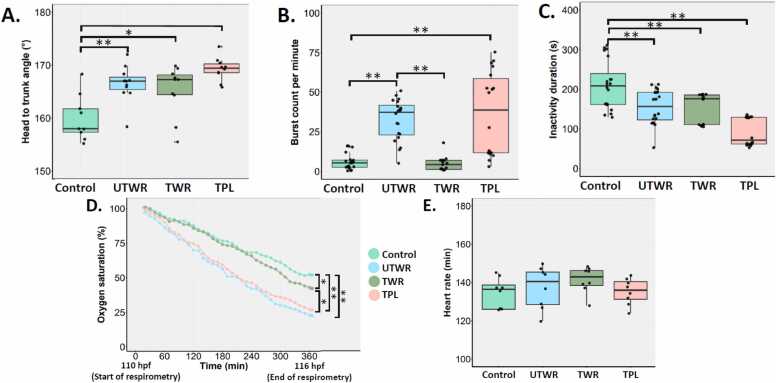
Effect of tyre-wear pollutants on early embryonic movement, respiration and heart rate in zebrafish larvae. **A)** Head to trunk angle at 116 hpf, **B)** burst count per min at 24 hpf, **C)** inactivity duration at 24 hpf, **D)** oxygen levels between 110 and 116 hpf and **E)** heart rate at 116 hpf. Statistical significance is indicated by * (p < 0.05) and ** (p < 0.005).

The body length of the zebrafish larvae was also measured at 72, 96 and 116 hpf (Supplementary Fig. 1B**-D**), but the only significant effect was observed at 116 hpf in TWR-exposed larvae (Supplementary Fig. 1B**-D**; p < 0.005). Also, a significant reduction in egg size was observed at 24 hpf in the eggs exposed to UTWR, TWR, and TPL compared to the control (Fig. 2**B**; p < 0.005). Additionally, egg size was significantly reduced in the eggs exposed to UTWR compared to those exposed to TWR (Fig. 2**B**; p < 0.005). The eye size in the exposed larvae was measured at 72, 96, and 116 hpf. We did not observed significant changes in eye size in larvae exposed to any treatment group when compared to control group (Fig. 2**C**)The size of the yolk sac (96, 116 hpf) and swim bladder (116 hpf) was also measured (Supplementary Fig. 1F**-H**), but no significant effect was observed in any of the treatment groups when compared to the control (p > 0.05). We also observed developmental deformities such as jaw deformity, uninflated swim bladder, reduced yolk resorption, yolk sac edema and pericardial edema in larvae exposed to UTWR, and TPL (Fig. 2**D**). Head to trunk angle was significantly increased in the larvae exposed to UTWR, TWR and TPL (Fig. 3**A**).

### 3.4 Compromised early embryonic movement at 24 hpf

A significant increase in burst count per min was observed in the zebrafish larvae exposed to the UTWR and TPL (Fig. 3**B**; p < 0.005) compared to the control. Also, there was a significant increase in burst count in the larvae exposed to UTWR compared to the TWR (Fig. 3**B**; p < 0.005). However, TPL exposure to larvae had no significant effect on their burst count (Fig. 3**B**; p > 0.005). A significant decrease in the inactivity duration was also observed in larvae exposed to any of the four treatments compared to the control (Fig. 3**C**; p < 0.005).

### 3.5 Increased oxygen consumption at 113 and 116 hpf

At 113 hpf, TWR exposure did not change oxygen consumption in the zebrafish larvae compared to the control group (Fig. 3**D**; p > 0.05). However, a significant increase in oxygen consumption was observed at 116 hpf (Fig. 3**D**; p < 0.05). Conversely, UTWR exposure significantly increased oxygen consumption at 113 and 116 hpf compared to the control group (Fig. 3**D**; p < 0.05). Furthermore, the UTWR-exposed larvae consumed significantly more oxygen than the TWR-exposed larvae (Fig. 3**D**; p < 0.05). The TPL exposure also resulted in significantly decreased oxygen levels in the water at 113 hpf and 116 hpf compared to the control (Fig. 3**D**; p < 0.005).

### 3.6 No effect on heart rate

A statistically significant effect of exposure on heart rate was not observed in any treatment group compared to the control group (Fig. 3**E**; p > 0.05). We observed pericardial edema in few larvae exposed to TPL (Fig. 2**D**); however, there was no significant change in heart size in the same group when compared to the control group.

### 3.7 Hypolocomotion in exposed larvae

**Fig. 4:**
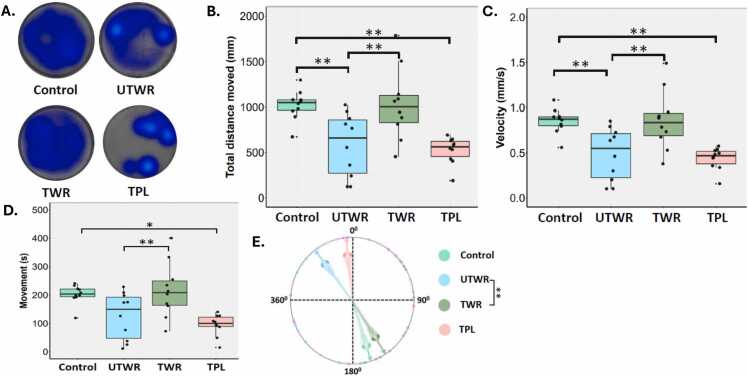
Effect of tyre-wear pollutants on swimming behaviour in zebrafish larvae at 116 hpf. **A)** Representative locomotory heatmaps, **B)** total distance moved, **C)** velocity, **D)** movement and **E)** angular velocity. Statistical significance is indicated by * (p < 0.05) and ** (p < 0.005).

Hypolocomotion or decreased locomotory activity was observed in the larvae exposed to UTWR and TPL when compared to the control, as seen on the representative locomotory heatmaps (Fig. 4**A**). Exposure to UTWR and TPL significantly reduced the distance, velocity and movement of the zebrafish larvae when compared to the control (Fig. 4**B-D**; p < 0.005). Also, there was a significant decrease in locomotory parameters in larvae exposed to UTWR when compared to TWR (Fig. 4**B-D**; p < 0.005). Similarly, there was a significant difference in the angular velocity between TWR and UTWR (Fig. 4**E**; p<0.005).

### 3.8 Transcriptional modifications

The whole-larva RNA sequencing generated 386.6 million raw reads, with an average of 19.33 million reads per sample (Supplementary Table 3). Of these total raw reads, 98.9 % reads were of good quality (Supplementary Table 3). Good-quality reads were mapped to reference the zebrafish genome, and the average mapping rate was around 92.78 % (Supplementary Table 3). Principal component analysis (PCA) was performed, and a plot was prepared to explore the variation between different treatment groups (Supplementary Fig. 2A). UTWR, TWR, and TPL groups exhibited overlapping confidence ellipses, indicating that these treatments shared more similarities in their responses (Supplementary Fig. 2A). Despite the overlap, these groups were still distinguishable from each other and the control group (Supplementary Fig. 2A). The general distribution plot of DEGs was prepared to assess the spread of up-and down-regulated DEGs in each treatment (Supplementary Fig. 2B). When comparing the control group and UTWR, a total of 687 DEGs were identified, with 52 genes upregulated and 635 downregulated (Supplementary Table 4). Similarly, a comparison of the control group and TWR revealed 392 DEGs, split with 66 upregulated and 326 downregulated DEGs (Supplementary Table 5). In the TPL group, 962 DEGs were generated, with 91 upregulated and 871 downregulated (Supplementary Table 6). Based on the number of significant DEGs related to detoxification, the degree of downregulation was more pronounced in UTWR-exposed larvae than in TWR-exposed larvae (UTWR; 32 DEGs, TWR; 17 DEGs), p-adjusted value (UTWR; 0.023, TWR; 0.034) and log2 fold change (UTWR; −1.43, TWR; −1.40).

**Fig. 5:**
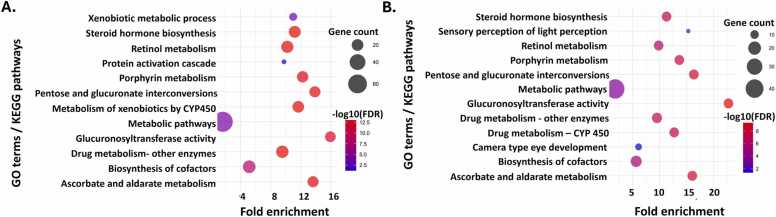
Gene Ontology (GO) terms and Kyoto Encyclopedia of Genes and Genomes (KEGG) pathway analysis of significant genes in zebrafish larvae exposed to tyre-wear pollutants. Transcriptomic analysis of zebrafish larvae exposed to tyre-wear pollutants. **A)** GO terms and KEGG pathway analysis of downregulated DEGs in zebrafish larvae exposed to untreated tunnel wash runoff (UTWR) and **B)** GO terms and KEGG pathway analysis of downregulated DEGs in zebrafish larvae exposed to treated tunnel wash runoff (TWR). The x-axis shows the fold enrichment, and the y-axis lists the significantly affected GO terms or KEGG pathways. The size of the circles represents the gene count, while the color indicates the –log10 of false discovery rate (FDR).

**Fig. 6:**
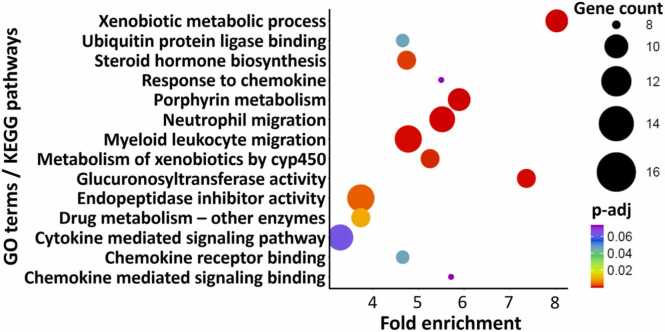
Gene Ontology (GO) terms and Kyoto Encyclopedia of Genes and Genomes (KEGG) pathway analysis of significant genes in zebrafish larvae exposed to tyre-particle leachate (TPL), The x-axis shows the fold enrichment, and the y-axis lists the significantly affected GO terms or KEGG pathways. The size of the circles represents the gene count, while the color indicates the padj value.

GO terms and KEGG pathway analysis were performed to understand the molecular pathways behind UTWR, TWR and TPL exposure to zebrafish larvae. In the UTWR-exposed larvae, 12 pathways were downregulated, and these were mainly related to the metabolism of xenobiotics, steroid hormone synthesis and metabolic pathways (Supplementary Table 7**;** Fig. 5**A**). In the TWR-exposed larvae, 15 pathways were downregulated, predominantly involving glucuronidation and glycosylation processes, as well as xenobiotic and drug metabolism (Supplementary Table 8**;** Fig. 5**B**). Many significantly affected pathways in UTWR and TWR-exposed larvae were similar. However, the pathways in TWR-exposed larvae were more significantly enriched in terms of p-adj value and FDR. TPL exposure led to the downregulation of 22 pathways in the larvae and these were related to xenobiotic metabolism, immune suppression, drug metabolism and enzyme activity (Supplementary Table 9**;** Fig. 6).

## 4 Discussion

Tunnel wash runoff refers to the wastewater generated from cleaning road tunnels. Estimating the actual amount of runoff generated and released into the environment is difficult since it depends on many factors, such as frequency of cleaning, amount of water used and vehicular traffic. This runoff contains a mixture of pollutants such as heavy metals, PAHs, PCBs, PFAS and tyre chemicals [51]. In the present study we found high concentrations of Fe, Cu, Ni, Zn, PAHs and aliphatic hydrocarbons in UTWR, TWR and TPL samples. However, the concentration of most of the heavy metals were below their known lethal limits and exposure solutions were also diluted (25 % UTWR and 50 % TWR). For example, for Hg, the detected levels were below 0.2 µg/L in the UTWR, TWR and TPL samples. Whereas known effect concentration on hatching time and survivability is 1.2 µg/L Hg in zebrafish eggs and larvae [7]. According to the OECD guidelines, up to 10 % mortality within 120 hpf is generally considered normal or insignificant [36]. In the main exposure study, we used 25 % UTWR and 2 g/L TPL as exposure dose and the mortality of larvae observed at 96 hpf was approximately 25 % in both the treatment groups. One probable reason for the observed mortality could be mixture toxicity of various heavy metals and organic chemicals. Also, the chemical composition varied between the different exposures, with TPL samples dominated by a very high concentration of Zn (6.27 mg/L), orders of magnitude above that observed in UTWR. The presence of extremely high amount of Zn in the TPL could be the reason behind significant mortality in this group. The higher Ni concentration in TWR than in UTWR could be due to fact that, unlike Fe or Zn which precipitate out as hydroxides or oxides, Ni stays soluble over a wider pH range, making it harder to remove unless specific adsorbents are used [54]. Additionally, leaching from treatment system components (pipes, tanks or fittings) or desorption of Ni from filtration media could have contributed to the increased levels observed post-treatment. Among quantified organic chemicals, TPL had a relatively higher concentration of low molecular weight aromatics while the aromatics detected in UTWR had a higher molecular mass. This could be due to depletion of semi-volatile organic compounds in the UTWR resulting from environmental conditions within the tunnel or due to their emission directly from the tyre surface during use while driving. Previous studies [14], [26] have identified larger PAHs in TP and TPL, and they may have been overlooked in the current study due to detection limits. The higher concentration of 6PPDq in the UTWR compared to TPL may reflect the rapid oxidation of 6PPD once released from vehicle tyres and a high degree of persistency. The low 6PPDq values in TWR indicate that the treatment process can remove a high proportion of this important chemical. Exposure to UTWR runoff and TPL led to increased mortality, impaired growth, developmental disorders, altered swimming behaviour and transcriptional dysregulation.

A reduction in egg size, body length and eye size in larvae exposed to UTWR, TWR and TPL was observed, which aligns with many previously published studies. In a study by Skarsjø [41], exposure to UTWR significantly reduced the growth of juvenile brown trout (*Salmo trutta*). Chibwe et al. [6] observed a decrease in the body length of flathead minnow (*Pimmephales promelas*) exposed to 10 g/L TPL for ten days. In the present study, we identified five DEGs related to growth and development that were significantly downregulated across all treatment groups: *glud1a*, *ccnb1*, *mvp, gdf6*, and *nmnat1*. Glutamate dehydrogenase 1a (*glud1a*) is crucial for amino acid metabolism and energy production [22], while cyclin B1 (*ccnb1*) plays a crucial role in cell cycle progression [9]. Similarly, growth differentiation factor 6 (*gdf6*) plays a pivotal role in skeletal and ocular development, and disruptions in its expression could explain the reduced eye size [2]. This suggests that UTWR, TWR and TPL may trigger similar mechanisms, causing oxidative stress and dysregulation of genes associated with growth and apoptosis. However, the intensity of downregulation of these genes varies according to pollutant exposure. Based on log2fold change and padj values, the severity of the effect was in the following order: TPL>UTWR>TWR. Also, the phenotypic responses (reduced body length, egg size, and eye size) were reflected by the transcriptomic data.

Larvae exposed to UTWR, TWR and TPL exhibited a significant increase in oxygen consumption. None of the previously published studies have specifically examined the effects of UTWR, TWR, and TPL exposure on respiration rate or oxygen consumption. The transcriptomics findings pointed to downregulation of the porphyrin metabolic pathway, which could be the reason behind the increased oxygen consumption in larvae exposed to UTWR, TWR and TPL. The porphyrin metabolic pathway is involved in the synthesizing of heme, a vital molecule involved in cellular respiration [28], [57]. Also, we identified three common DEGs dysregulated among all treatment groups and related to mitochondrial function (*polg*) and energy metabolism (*glud1a* and *nmnat1*), which could be likely candidates for influencing oxygen consumption in zebrafish larvae. The *polg* gene encodes the catalytic subunit of mitochondrial DNA polymerase gamma, responsible for the replication and repair of mitochondrial DNA. In contrast, the *glud1a* and *nmnat1* (nicotinamide mononucleotide adenylyl transferase) genes encode proteins involved in the conversion of metabolic intermediates and cofactors, which are essential in cellular respiration and ATP production [22], [29]. Detoxification pathways play a key biological role by making toxic substances less toxic [55]. Enzymes like cytochrome P450s (CYPs), glutathione S-transferases (GSTs), and UDP-glucuronosyltransferases (UGTs) contribute to neutralizing and eliminating xenobiotic compounds. In the present study, we observed downregulation of several CYP, GST and UGT genes in larvae exposed to UTWR and TWR, probably reflecting a disruption in the zebrafish's larvae ability to process and eliminate these contaminants.

Significant adverse effects were found on embryonic movement and swimming behaviour in larvae exposed to UTWR, TWR and TPL. The results from the present study align with several previously published studies that observed neurotoxic and locomotory effects of TPL in fish [24], [4]. We found five DEGs (*dclk2b*, *gripap1*, *unc45a*, *kcnh2* and *casq2*) that were common across the treatments, encoding proteins having neurological function that might be linked to locomotory behaviour. Several studies have shown that these genes encode proteins involved in neuronal signalling and synaptic function [40], [44], [53]. The downregulatory effect on these genes can lead to reduced neuronal excitability and impaired synaptic transmission, ultimately leading to altered swimming behaviour. Furthermore, dysregulation of genes associated with muscle contraction, such as *unc45a* (unc-45 myosin chaperone A) and *casq2* (calsequestrin 2), suggests that exposure to UTWR, TWR and TPL also impairs mechanisms linked to muscular function, contributing to the observed behavioural dysfunctioning in the zebrafish larvae. Taken together, the strongest neuro and behavioural toxicity were observed in embryos exposed to TPL, followed by UTWR and TWR, respectively.

The results from our study suggest that chemicals, including those leaching from tyres, are likely to be the drivers of toxicity in tunnel-wash runoff. Elevated levels of several tire-chemicals including heavy metals (such as Zn and Cu), PAHs, and 6PPDq in UTWR appear to contribute significantly to the toxicity observed in zebrafish larvae, as evidenced by increased mortality, impaired growth, and altered swimming behaviour. Ji et al. [18] found that 6PPDq exposure reduced spontaneous movement in zebrafish embryos, indicating neurodevelopmental toxicity. The high concentrations of 6PPDq, along with other pollutants in UTWR compared to TWR, suggest that it may contribute to UTWR toxicity. This is corroborated by increased oxygen consumption, hypolocomotion, and transcriptional alterations in larvae exposed to UTWR. Overall, the main finding of this study reflects that although the treatment process has reduced the toxicological burden on larval physiology, they are insufficient to fully mitigate the harmful effects. Hence, improved treatment practices are needed to further reduce the levels of tunnel wash runoff contaminants in the environment.

In addition to the observed effects on zebrafish larvae, wider environmental consequences of tunnel runoff should be considered. The release of large volumes of UTWR can have significant ecological effects, particularly in nearby smaller creeks and aquatic ecosystem. For example, an episode of acute death of Atlantic salmon (*Salmo salar*) happened after tunnel washing in the Homla River near the Stavsjøfjell Tunnel on the E6 highway in Norway in 2018 [39]. However, it was not evident what led to mass mortality of salmon in the Homla River. Tire-related compounds, including degradation byproducts, have previously been found to cause similar effects and cannot be ruled out as contributing factors in such events. Similarly, in Washington’s Puget Sound, stormwater pollution has resulted in pre-spawn mortality of Coho salmon [46]. These event highlights the vulnerability of aquatic organisms to such pollutants. Smaller waterways and their ecosystems are particularly susceptible, as they lack the buffering capacity of larger systems. These findings underscore the urgent need to address both particle runoff and chemical contaminants from tunnel wash water through enhanced treatment processes and regulatory measures to safeguard aquatic biodiversity.

## 5 Conclusions

This is the first study to document the difference in toxicity between UTWR and TWR, as well as to compare the toxicity to chemicals leaching from vehicle tyres. The filtration process reduced the levels of most contaminants, but diluted TWR can still be toxic to early life stages of fish. Notably, the chemical composition of TPL differed substantially from that of UTWR, with very few compounds in common, suggesting that non-tyre-wear sources also contribute to the toxicity of TWR. Zebrafish embryos exposed to these contaminants showed developmental impairments, while larvae exhibited abrupted swimming behaviour, and increased oxygen consumption. The transcriptomic data supported the phenotypic findings. In conclusion, further investigation is needed on how to reduce the levels of contaminants in TWR before release into nature and how to mitigate the impacts of tyre-derived pollutants on aquatic ecosystems.

## CRediT authorship contribution statement

**Prabhugouda Siriyappagouder:** Methodology. **Chinmayi Ramaghatta:** Writing – review & editing, Writing – original draft, Methodology, Data curation. **Lisbet Sørensen:** Writing – review & editing, Methodology. **Andy M. Booth:** Writing – review & editing, Methodology. **Olsvik Pål A:** Writing – review & editing, Supervision, Conceptualization. **Shubham Varshney:** Writing – review & editing, Writing – original draft, Methodology, Data curation, Conceptualization.

## Declaration of Competing Interest

The authors declare that they have no known competing financial interests or personal relationships that could have appeared to influence the work reported in this paper.

## Data Availability

Data will be made available on request.
