# Supplementary material for: Multi-endpoint assessment of tunnel wash water and tyre-particle leachate in zebrafish larvae
Source: Toxicol Rep. 2025 Jul 23;15:102096. doi: 10.1016/j.toxrep.2025.102096 (PMC12318315; doi:10.1016/j.toxrep.2025.102096)
Supplement: Supplementary file 1 — Supplementary material [file mmc1.pdf]

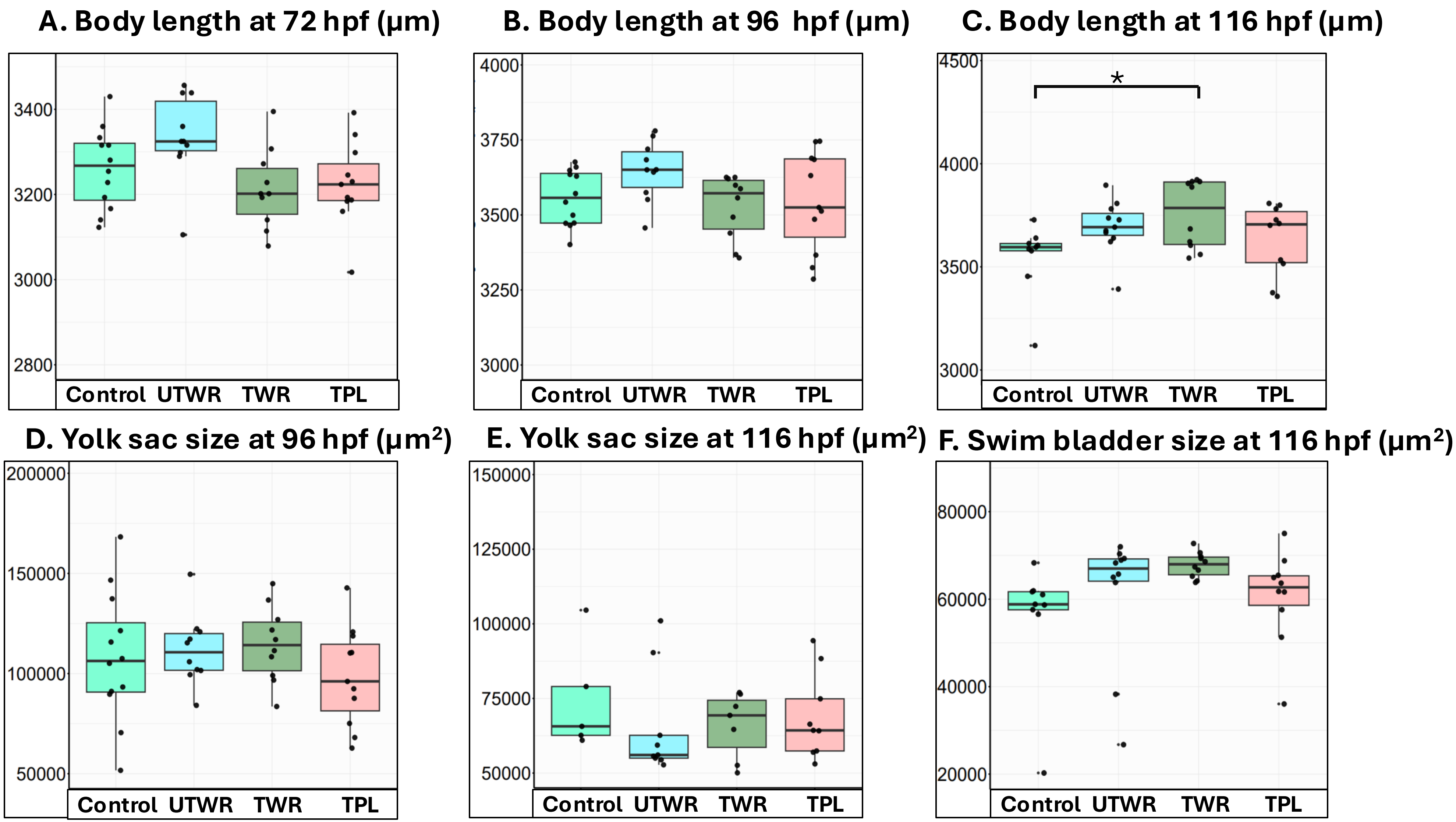

Supplementary figure 1. Effect of tyre-wear pollutants on the developmental indices of zebrafish larvae. **A-C)** body length measurements at 72, 96, and 116 hpf, respectively, **D-E)** yolk sac size at 96 hpf and 116 hpf, respectively and **F)** swim bladder size at 116 hpf. Statistical significance is indicated by * ($p < 0.05$) and ** ($p < 0.005$).

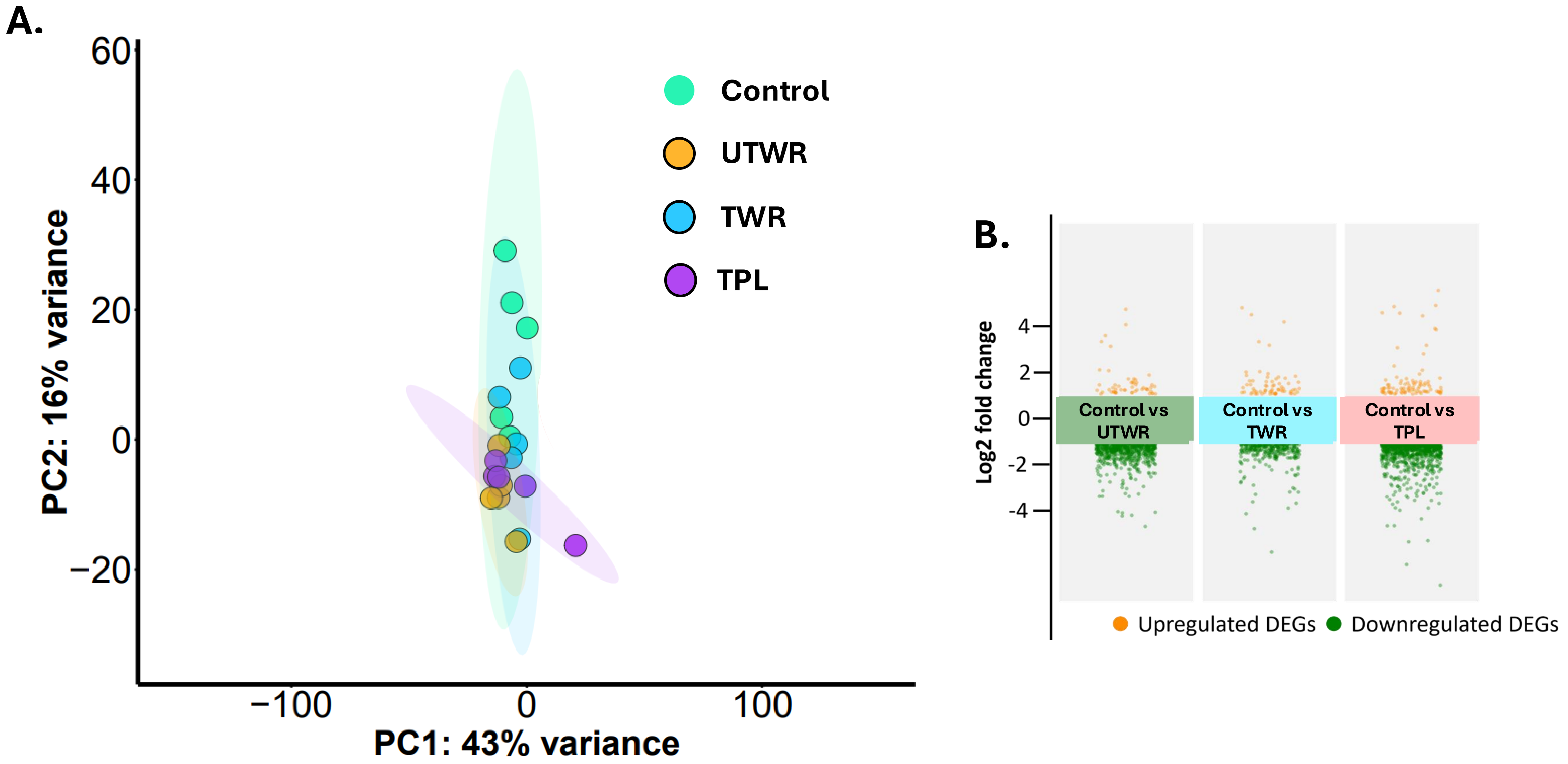

Supplementary figure 2. Transcriptomic analysis of zebrafish larvae exposed to tyre-wear pollutants. **A)** Principal component analysis (PCA) plot and **B)** general distribution plot of differentially expressed genes (DEGs).
